# Supplementary material for: Identification of photoperiod-induced specific miRNAs in the adrenal glands of Sunite sheep (Ovis aries)
Source: Front Vet Sci. 2022 Jul 22;9:888207. doi: 10.3389/fvets.2022.888207 (PMC9354845; doi:10.3389/fvets.2022.888207)
Supplement: Supplementary file 1 [file Data_Sheet_1.ZIP › Supplementary file/miRDeep2 file.zip!/miRDeep2 file/Know_Structure/LP42A3/oar-mir-376b.pdf]

miRBase precursor : oar-mir-376b
Total read count : 740
oar-miR-376e-5p read count : 278
oar-miR-376b-5p read count : 278
oar-miR-376b-3p read count : 184
remaining reads : 0

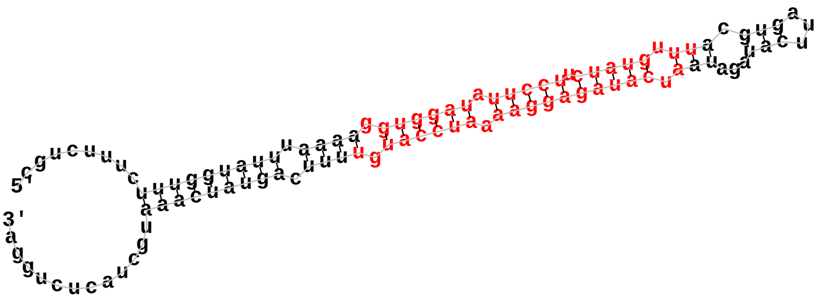

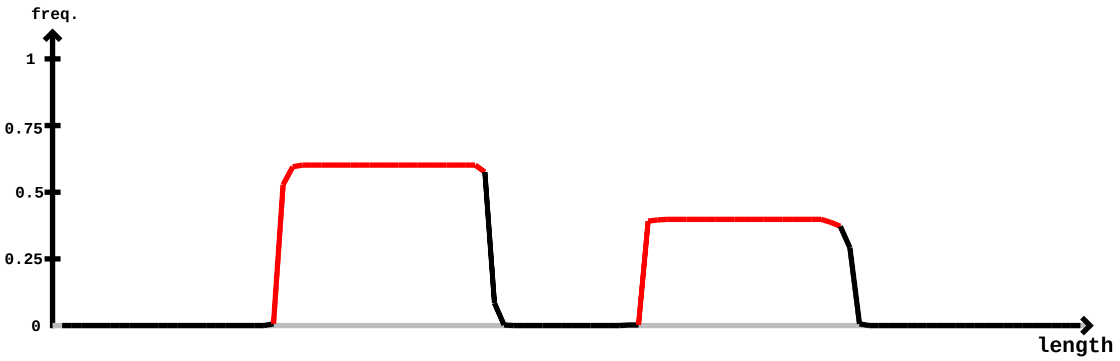


```
                                                            oar-miR-376b-3p
                      oar-miR-376b-5p
                      oar-miR-376e-5p
5'- cgucuuucuuugguauuuaaaaggugggauauuccuucuauguuuacgugauucauagauaaucauagaggaaaauccauguuuucaguaucaaaugcuacucugga   -3'    exp
    ........(((((((((.((((.((((((.(((((.(((((.(((.(((...)))...))).)))))))))).)))))).)))).)))))))))............    reads      mm          sample
    .....................agguggauauuccuucuauguuu..............................................................    3          0           seq
    ......................gguggauauuccuucuauguu...............................................................    11         0           seq
    ......................gguggauauuccuucuauguuu..............................................................    197        0           seq
    ......................gguggauauuccuucuauguuua.............................................................    32         0           seq
    ......................gguggauauuccuucuauguuuac............................................................    1          0           seq
    .......................guggauauuccuucuauguu...............................................................    1          0           seq
    .......................guggauauuccuucuauguuu..............................................................    25         0           seq
    .......................guggauauuccuucuauguuua.............................................................    5          0           seq
    ........................uggauauuccuucuauguuu..............................................................    2          0           seq
    ........................uggauauuccuucuauguuua.............................................................    1          0           seq
    ..........................................................uaaucauagaggaaaauccaug..........................    1          0           seq
    ............................................................aucauagaggaaaauccau...........................    5          0           seq
    ............................................................aucauagaggaaaauccaug..........................    6          0           seq
    ............................................................aucauagaggaaaauccaugu.........................    37         0           seq
    ............................................................aucauagaggaaaauccauguu........................    129        0           seq
    ............................................................aucauagaggaaaauccauguuu.......................    3          0           seq
    .............................................................ucauagaggaaaauccauguu........................    2          0           seq
    ..............................................................cauagaggaaaauccauguu........................    1          0           seq
```
